# Injectable thermo-sensitive hydrogel loaded hollow copper sulfide nanoparticles for ROS burst in TME and effective tumor treatment

**DOI:** 10.3389/fbioe.2023.1191014

**Published:** 2023-05-02

**Authors:** Shipeng Ning, Jianlan Mo, Rong Huang, Benkun Liu, Bicheng Fu, Shuaijie Ding, Huawei Yang, Ying Cui, Lei Yao

**Affiliations:** ^1^ Guangxi Medical University Cancer Hospital, Nanning, China; ^2^ Department of Anesthesiology, Maternal and Child Health Hospital of Guangxi Zhuang Autonomous Region, Nanning, China; ^3^ Department of Thoracic Surgery, Harbin Medical University Cancer Hospital, Harbin, China; ^4^ Department of Gastrointestinal Surgery and Department of Geriatrics, Shenzhen People’s Hospital (The Second Clinical Medical College, Jinan University, The First Affiliated Hospital, Southern University of Science and Technology), Shenzhen, Guangdong, China; ^5^ Department of Radiation Oncology, Harbin Medical University Cancer Hospital, Harbin, China

**Keywords:** lung cancer, hydrogel, *β*-lapachone, CDT, self-supplies H_2_O_2_

## Abstract

**Introduction:** Lung cancer the most prevalent cause of cancer-related deaths, and current therapies lack sufficient specificity and efficacy. This study developed an injectable thermosensitive hydrogel harboring hollow copper sulfide nanoparticles and β-lapachone (Lap) (CLH) for lung tumor treatment.

**Methods:** The hydrogel-encapsulated CLH system can remotely control the release of copper ions (Cu^2+^) and drugs using photothermal effects for non-invasive controlled-release drug delivery in tumor therapy. The released Cu^2+^ consumes the overexpressed GSH in TME and the generated Cu^+^ further exploits the TME characteristics to initiate nanocatalytic reactions for generating highly toxic hydroxyl radicals. In addition, in cancer cells overexpressing Nicotinamide adenine dinucleotide (phosphate): quinone oxidoreductase 1 (NQO1), Lap can catalyze the generation of hydrogen peroxide (H_2_O_2_) through futile redox cycles. H_2_O_2_ is further converted into highly toxic hydroxyl radicals via the Fenton-like reaction, leading to a burst of reactive oxygen species in TME, which further enhances the therapeutic effect of chemokines.

**Results:** Analysis of the antitumor efficacy in a subcutaneous A549 lung tumor model mice showed a significant delay in tumor growth and no systemic toxicity was detected.

**Discussion:** In conclusion, we have established a CLH nanodrug platform that enables efficient lung tumor therapy through combined photothermal/chemodynamic therapy (CDT) treatment and self-supplying H_2_O_2_ to achieve cascade catalysis, leading to explosive amplification of oxidative stress.

## Introduction

In recent years, the incidence of lung cancer has been increasing steadily, with mortality rates for lung cancer remaining high (Sugarbaker and DaSilva, 2011). Non-small cell lung cancer (NSCLC) accounts for approximately 80% of lung cancer, of which early NSCLC accounts for only 20%–30%, and surgery is the main treatment (Milano et al., 2014; Evison et al., 2015; Vallone et al., 2017). However, the adverse reactions of chemotherapy drugs limit wide clinical application, so it is paramount to develop a novel lung cancer therapeutic option. Being an emerging and effective cancer treatment strategy, chemodynamic therapy (CDT) has recently attracted a lot of attention in the field of cancer treatment (Yu et al., 2021; Deng et al., 2022; Peng et al., 2023). It converts weakly oxidized hydrogen peroxide (H_2_O_2_) into highly cytotoxic hydroxyl groups (·OH) *in situ* within tumor region via a Fenton/Fenton-like reaction, thereby inducing apoptosis and inhibiting tumor growth (Shen et al., 2018; Dong et al., 2019). In contrast to conventional therapies, CDT is not dependent on intracellular oxygen content and does not require exogenous energy such as X-rays, continuous light and ultrasound input, which not only effectively avoids the limited penetration depth of light and radiation from X-rays, but also overcomes the inherent barrier of hypoxia within tumor area. In addition, CDT is less toxic to normal cells and can achieve specific killing of tumor cells, and therefore has great potential for application in tumor therapy. However, CDT research is still in its infancy and the therapeutic effect is not yet ideal. Methods for effectively improving the therapeutic effect of CDT is still an important research direction (Yu et al., 2020; Yang et al., 2021). In addition, GSH overexpressed within tumor microenvironment (TME), as an important antioxidant in cells, can scavenge the generated hydroxyl radicals (·OH) and weaken the cellular redox effect (Zhu et al., 2018; Zhu et al., 2022a), and this antioxidant defense of cancer cells becomes a major obstacle for CDT effectiveness (Franco et al., 2007; Chang et al., 2019; Wu et al., 2020).

At present, research on the use of nanomedicines for tumor treatment is emerging endlessly (Zhu et al., 2020; Zhu et al., 2021a; Chen et al., 2022a; Dai et al., 2022; Li et al., 2022; Opoku‐Damoah et al., 2022; Xiang et al., 2022; Cao et al., 2023; Lu et al., 2023; Ning et al., 2023). Iron-based nanomaterials were widely used as classical Fenton reaction catalysts in CDT studies (Feng et al., 2020; Cheng et al., 2021). However, reaction of Fe^2+^-mediated Fenton reaction is relatively inefficient and strongly dependent on the acidic environment, thus the reaction efficiency is low within weakly acidic TME, resulting in slow generation of reactive oxygen species and limited therapeutic effects (Lin et al., 2020). In recent years, it has been found that besides Fe^2+^ catalyzing the decomposition of H_2_O_2_ to produce OH, other transition metal ions such as Mn^2+^, Cu^2+^ and Co^2+^ could also accelerate or replace Fe^2+^ to play this role (Liu et al., 2018; Fu et al., 2019; Sang et al., 2020). Among these metal elements, Cu^2+^ is renowned for its excellent properties. Firstly, as a cofactor of many natural enzymes in living organisms, copper has excellent biocompatibility and is widely involved in biochemical reactions *in vivo*. Moreover, it has been reported within literature that copper ion-like Fenton reactions have a broader pH range than Fe^3+^. More importantly, the conversion of Cu^2+^ to Cu^+^ can effectively consume intracellular GSH and reducing loss of ROS, with reduced Cu + reacting with H_2_O_2_ in tumor cells to produce OH, thus improving CDT efficiency and enhancing anti-tumor effects (Wu et al., 2019a; Ma et al., 2019). CuS, as a naturally occurring inorganic mineral, is not only capable of releasing Cu^2+^ under acidic conditions, though it can also react with H_2_O_2_ in tumor sites to produce ROS for CDT (Liu et al., 2021). Moreover, as one of the first-developed inorganic semiconductor photothermal reagents, CuS nanoparticles are able to convert irradiated near-infra-red (NIR) light into energy through local plasmon resonance effect (Liang et al., 2019; Ding et al., 2022). By delivering CuS nanoparticles (NPs) to the tumor tissue, photothermal therapy (PTT) can be performed through thermal effects when the tumor is exposed to NIR laser irradiation. Therefore, the combination therapy of PTT and CDT, based on CuS nanoparticles, could achieve excellent synergistic therapeutic effects. In addition to the Fenton-like properties of the metastable metal itself, the Fenton reaction rate is also dependent on the concentration of the reactant substance, such as the concentration of H_2_O_2_ (Cun et al., 2022; Deng et al., 2022). Although the H_2_O_2_ content in tumor sites is higher than for normal tissue (Zhu et al., 2022b; Zhu et al., 2022c), the limited content still regulates the rate of Fenton-like reactions, thus limiting the therapeutic effect of CDT.

The natural compound β-lapachone (Lap), chemically known as 3,4-dihydro-2,2-dimethyl-2H-naphtho [1,2-b]-pyran-5,6-dione, belongs to the group of 1,2-naphthoquinones (Cun et al., 2022). Lap retains a wide range of biological and pharmacological effects, with its pharmacological actions including antibacterial, anti-inflammatory, anticancer, and anti-angiogenic. The main cytotoxic mechanism of Lap is through NADP(H)-quinone oxidoreductase l (NQ01) bioactivation, which generates reactive oxygen species through a quinone-hydroquinone-quinone redox cycle process. The consumption of 60 mol of NAD(P)H per mole of Lap in approximately 2 min generates >120 mol of equivalent H_2_O_2_ (Wang et al., 2019). However, low solubility of Lap in water (0.038 mg/mL), short plasma half-life (24 min), narrow therapeutic window and the tendency to develop methemoglobinemia beyond the window, all greatly limit its application.

**SCHEME 1:**
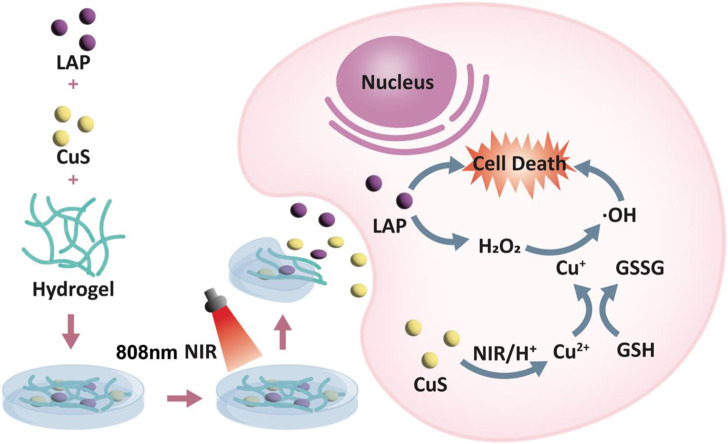
Schematic illustration of injectable thermo-sensitive hydrogel loaded hollow copper sulfide nanoparticles for reactive oxygen species burst within TME and attaining effective tumor treatment.

Herein, we have developed a CLH nanohydrogel system that amplifies oxidative stress through cascade catalysis by co-loading CuS nanoparticles and Lap into agarose hydrogels. The hydrogel delivery system can exist in the tumor site for a long time, avoiding the trouble and trauma of repeated injection in the tumor (Scheme 1) (Qiu et al., 2018; Wu et al., 2019b; Zhu et al., 2021b). This CLH system, with the excellent photothermal conversion efficiency of CuS NPs, is able to convert NIR light into thermal energy, leading to tumor warming and thus photothermal therapy, leading to tumor ablation. Agarose hydrogels has temperature sensitive characteristics (Qiu et al., 2018; Zhang et al., 2022), combined with the photothermal properties of CuS, to achieve demand-controlled Lap release. The acidic TME can accelerate the degradation of CuS NPs, and the released Cu^2+^ generates Fenton-like reaction with H_2_O_2_. Cu^2+^ can consume overexpressed GSH inside tumor tissue through redox reaction to generate Cu+, which further catalyzes H_2_O_2_ to generate cytotoxic OH radicals. Through this synergistic effect, intracellular ROS levels were significantly increased and oxidative stress was amplified. Meanwhile, the released Lap underwent a useless redox cycle in the presence of NQO1 and efficiently produced H_2_O_2_, which not only directly caused DNA damage, though also further increased the rate of Fenton-like reaction by self-supplying H_2_O_2_, thus enhancing the killing ability of CDT on tumor cells. *In vitro* and *in vivo* anti-tumor analysis of subcutaneous A549 lung tumors in murines showed that CLH significantly inhibited tumor growth, without adverse effects - such as inflammatory reactions. The system is combined with synergistic photothermal/chemical kinetics to induce H_2_O_2_ self-supply and achieve reactive oxygen species burst through cascade catalysis, thus achieving effective tumor treatment effects.

## Results and discussion

**FIGURE 1:**
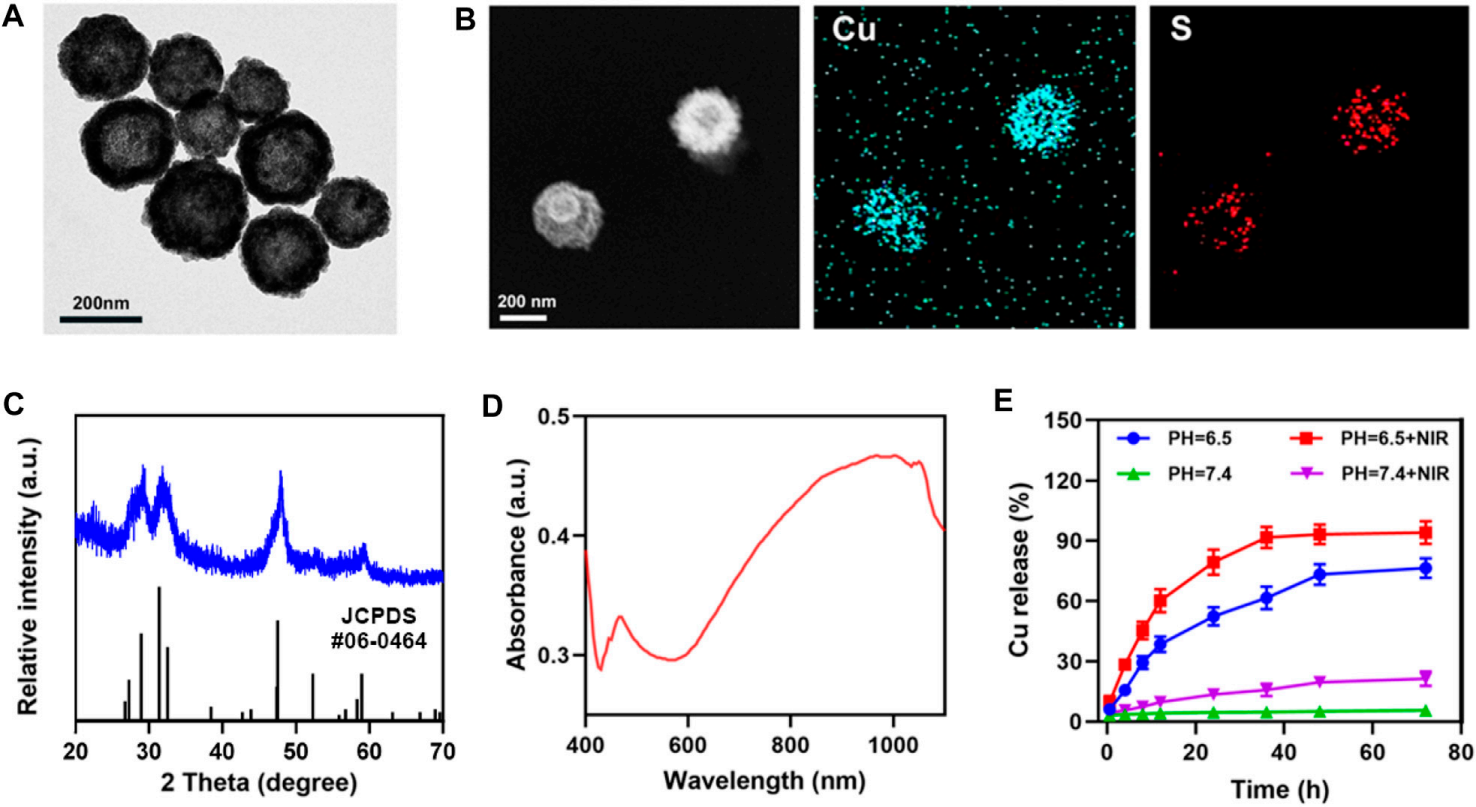
**(A)** TEM image, and **(B)** corresponding elemental mapping images of CuS. **(C)** XRD pattern of CuS. **(D)** CuS absorbance spectra. **(E)** Release profiles of copper ions in weak acidic and neutral environments, with or without irradiation (808 nm, 0.5 W cm^−2^).

Transmission electron microscopy (TEM) analysis revealed the morphology and size distribution of CuS NPs. A hollow nanostructure of CuS was observed. Studies have shown that this hollow CuS has higher photothermal conversion efficiency (30%) (Zhang et al., 2019) (Figure 1A). Their elemental mapping illustrates the Cu and S elements in CuS (Figure 1B). The powder X-ray diffraction (XRD) characterization also proved the phase structures of the obtained CuS nanocrystals. The patterns of obtained CuS Janus were consistent with the standard data of hexagonal phase CuS (JCPDS no. 06-0464) (Figure 1C). As shown in Figure 1D, the CuS dispersion exhibited a strong absorption band within NIR region, which could render the capability of CuS for photothermal conversion. Furthermore, this group examined the effect of laser in weak acidic and neutral environments on the release of copper ions, using inductively-coupled plasma optical emission spectrometry (Figure 1E), demonstrating that the 808 nm laser irradiation is capable of accelerating the release of copper ions in an acidic environment.

**FIGURE 2:**
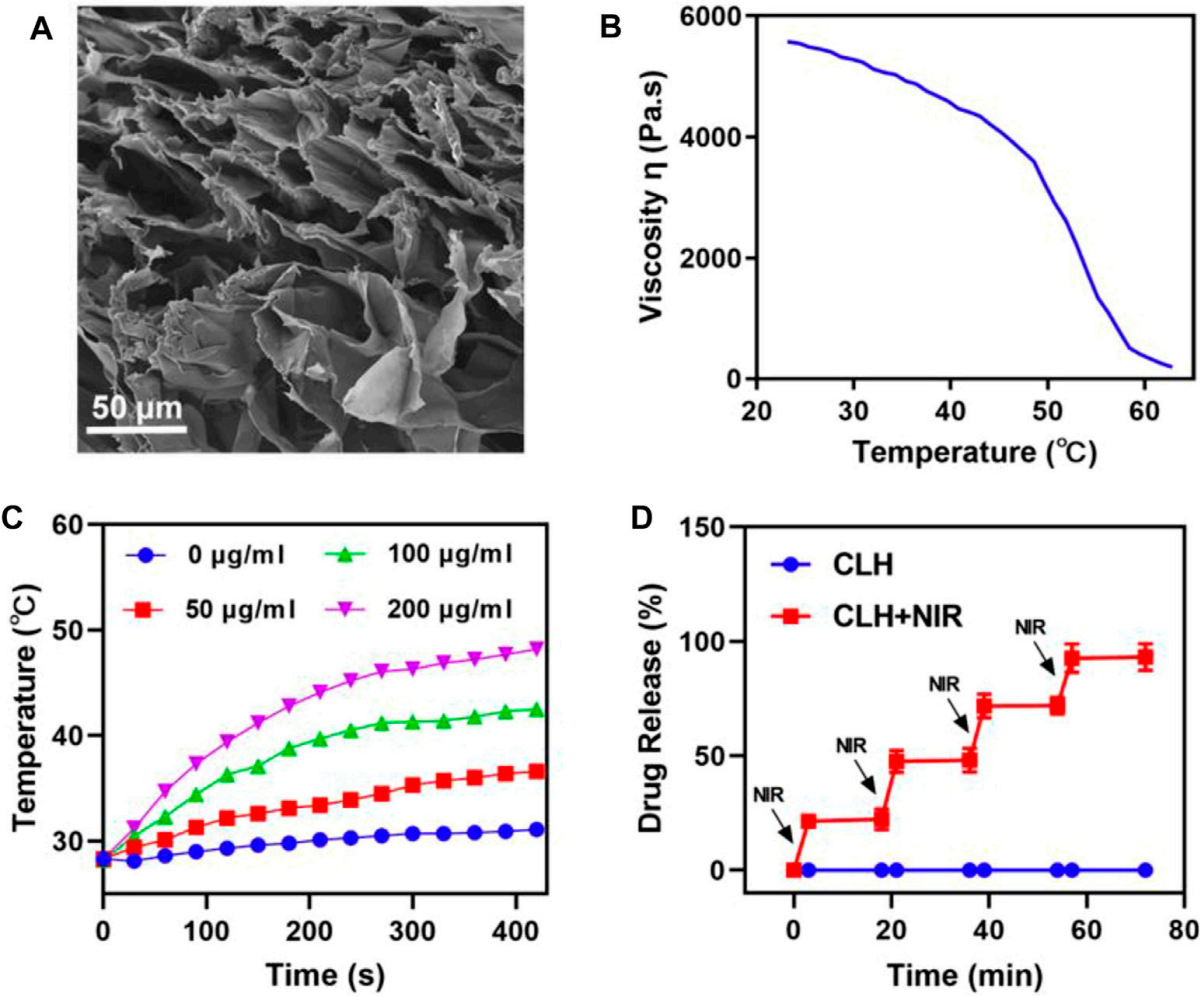
**(A)** Representative SEM images of CLH. **(B)** Viscosity measurements of reversibility for CLH during temperature jumps, from 23.17°C to 62.82°C. **(C)** Heating curves of varying CLH doses upon 808 nm laser at 0.5 W/cm^2^. **(D)** The CLH release profile, with or without 808 nm laser irradiation, with black arrows indicating irradiation time points.

The CLH platforms are prepared by co-encapsulating laplachones and preparing CuS NPs in FDA-approved agaroses, which are then characterized by scanning electron microscopy (SEM) (Figure 2A). Due to the elastic deformation ability of the hydrogel, it gradually dissolves on heating. When the temperature returns to ambient level, the hydrogel solidifies, consistent with the rheological curve within Figure 2B. Subsequently, the photothermal conversion properties of CLH hydrogel platforms, containing various doses of CuS NPs, were investigated by exposing them to a NIL-II laser for 7 minutes. It is worth noting that studies have shown that the safe laser power is lower than 0.6W cm^−2^, so it is appropriate for us to adopt 0.5 W cm^−2^ power here (Li et al., 2021). The dose- and time-dependent temperature rise curves were demonstrated (Figure 2C). The infrared thermal imaging technology further verifies that CLH has obvious temperature rise after irradiation, and also proves its excellent photothermal properties (Supplementary Figure S1). In addition, the experimental results also prove that CLH has good photothermal stability (Supplementary Figure S2). Combined with the excellent photothermal properties of CuS NPs, when irradiated with an 808 nm laser, the temperature of the hydrogel platform increased, resulting in liquefaction of the gel and consequent drug release (Figure 2D). Once the irradiation is halted, the temperature drops, the hydrogel solidifies, and release of the stored drug is halted, thus achieving CuS NPS-mediated photothermal controlled drug release/delivery.

**FIGURE 3:**
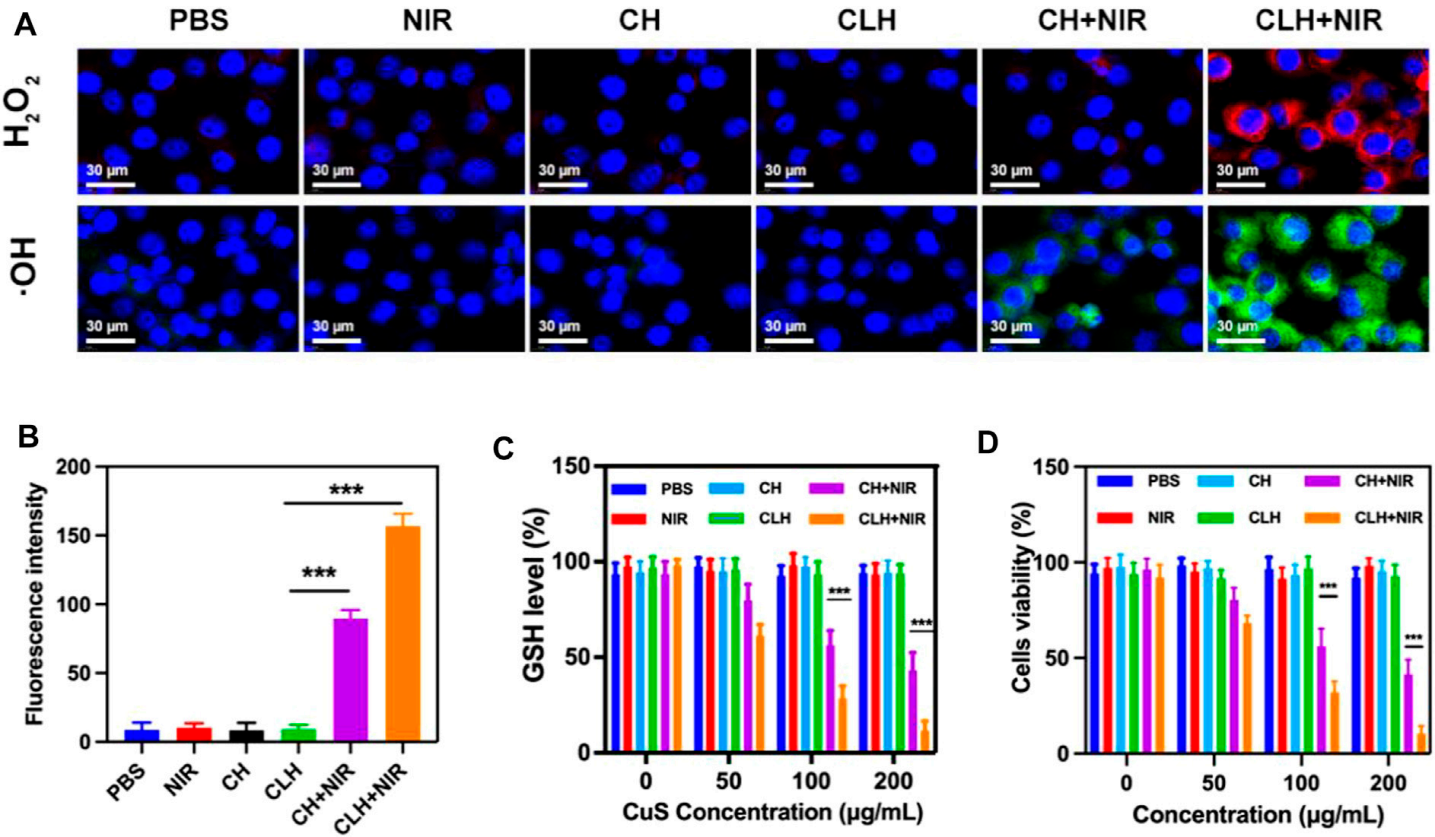
**(A)** Fluorescence images of ROS production in A549 cells following various treatments. **(B)** Fluorescence intensity of OH from Figure 3A. **(C)** The effect of different formulations on intracellular GSH levels (*n* = 3). **(D)** Cytotoxicity of differing doses of CuS NPs on A549 cells. (*n* = 3). *p < 0.05, **p < 0.01, ***p < 0.005; Student’s t-test.

Given the good photothermal effect of the CLH platform, this study subsequently explored the apoptotic effect of CLH *in vitro*. As mentioned previously, the main mechanism of tumor apoptosis by CLH could be the dissolution of hydrogel under light and the release of Lap. This latter event can catalyze the production of H_2_O_2_ through an ineffective redox cycle, in-turn catalyzed by the overexpression of NQO1 in tumor tissue. The generated H_2_O_2_ can not only cause direct tumor damage, though can also be further converted into highly toxic hydroxyl radicals via Fenton-like reactions, leading to the outbreak of reactive oxygen species within TME. In addition, Cu^2+^ can also deplete excess GSH within tumor region, further enhancing the production of ROS. Since ROS is a key factor within the induction of apoptosis by this CLH platform, this study first detected intracellular hydrogen peroxide and OH production in different treatment groups, using a hydrogen peroxide assay kit/hydroxyphenyl fluorescein (HPF). This study also prepared a CuS-coated hydrogel (CH) as a control group to further compare test results. The control group, laser group, CH group and CLH group alone hardly produced fluorescence signals (Figures 3A, B), probably since the drug could not function under the hydrogel wrapping. The combined CH + NIR group produced moderate OH fluorescence, though no H_2_O_2_ fluorescence was produced, indicating that the hydrogel dissolved under 808 nm laser irradiation and the released Cu^2+^ exerted the combined CDT and PTT. The CLH platform, constructed by adding Lap to the hydrogel platform, produced the strongest H_2_O_2_ fluorescence and OH fluorescence under the laser, which further demonstrated that lap-like can generate H_2_O_2_ within tumor region/s to further amplify the Fenton-like mediated oxidative stress. Typically, GSH is overexpressed within *in situ* tumor tissue to meet the redox homeostasis required for their growth, and its depletion of ROS is therefore considered a major obstacle to tumor therapy (Zhu et al., 2022a; Chen et al., 2022b; Opoku‐Damoah et al., 2022). To determine how the prepared CLH synergized with NIR radiation to deplete glutathione, this study examined GSH levels across differing treatment groups (Figure 3C). The GSH levels within CH + NIR and CLH + NIR groups were significantly decreased, possibly due to the depletion of intracellular GSH levels by Cu^2+^, while Lap could also produce a noticeable level of H_2_O_2_ to promote GSH depletion, further enhancing the therapeutic effect of CDT. Consequently, this study performed treatment combinations containing various doses of CuS on A549 tumor cells and assessed cellular viability through MTT assay (Figure 3D). Cells within control, laser and CLH treatment groups did not exhibit any significant cytotoxicity, which also indicated satisfactory biocompatibility for CLH. The CH + NIR group produced moderate cytotoxicity onto A549 cultures, whereas under 808 nm laser irradiation, CLH treatment produced significant cytotoxicity. Cell death was further enhanced with increasing CuS concentration, indicating that CLH-based apoptosis was concentration dependent. Thus, such assays demonstrated that this combined treatment strategy could compensate the deficiency of H_2_O_2_ within tumor mass, through Lap self-supply of H_2_O_2_ and depletion of GSH within tumor cells through Cu^2+^, thus further amplifying oxidative stress and improving the therapeutic effect. CuS can kill tumor cells significantly only at higher concentrations. On the one hand, it shows that CuS has good biological safety, and on the other hand, it suggests that direct injection of hydrogel into tumor to make it highly enriched may obtain better therapeutic effect.

**FIGURE 4:**
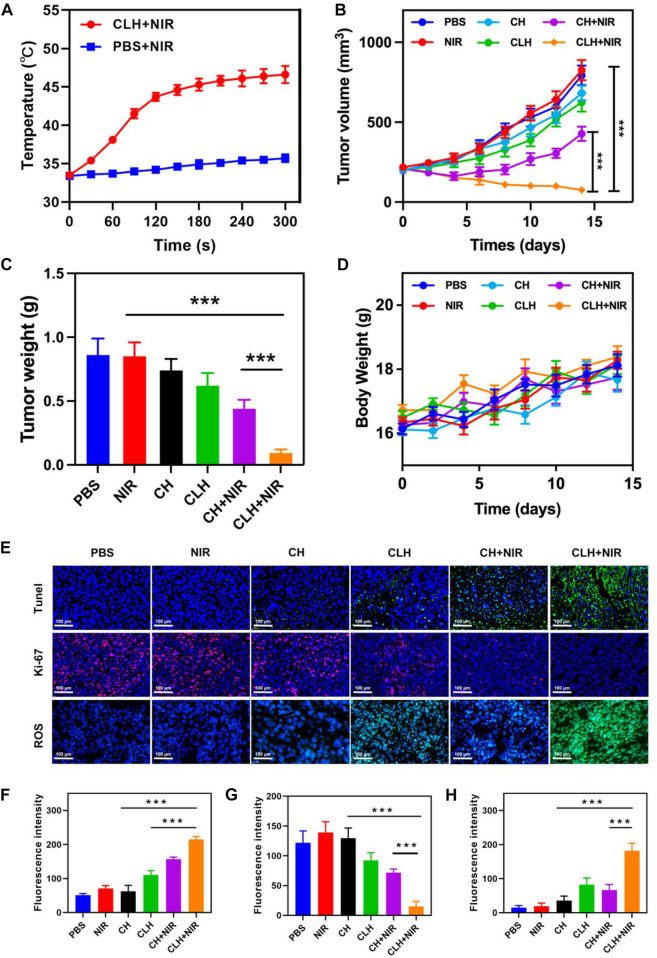
**(A)** Upon being irradiated with 808 nm laser at 0.5 W/cm^−2^ for 5 minutes, the temperature elevated in murines having A549 tumor, within specified treatment groups (*n* = 5). **(B)** Tumor-volume change curves of A549 tumor-bearing female BALB/C nude murines following various treatments (*n* = 5). **(C)** Tumor weight of A549 tumor-bearing female BALB/C nude murines following different treatments (*n* = 5). **(D)** Murine weight changes in different vivo treatments (*n* = 5). **(E)** TUNEL, Ki-67 and ROS immunofluorescence staining in tumor region of each group post-treatments. **(F)** TUNEL, **(G)** Ki-67 and **(H)** ROS fluorescence intensity.

**FIGURE 5:**
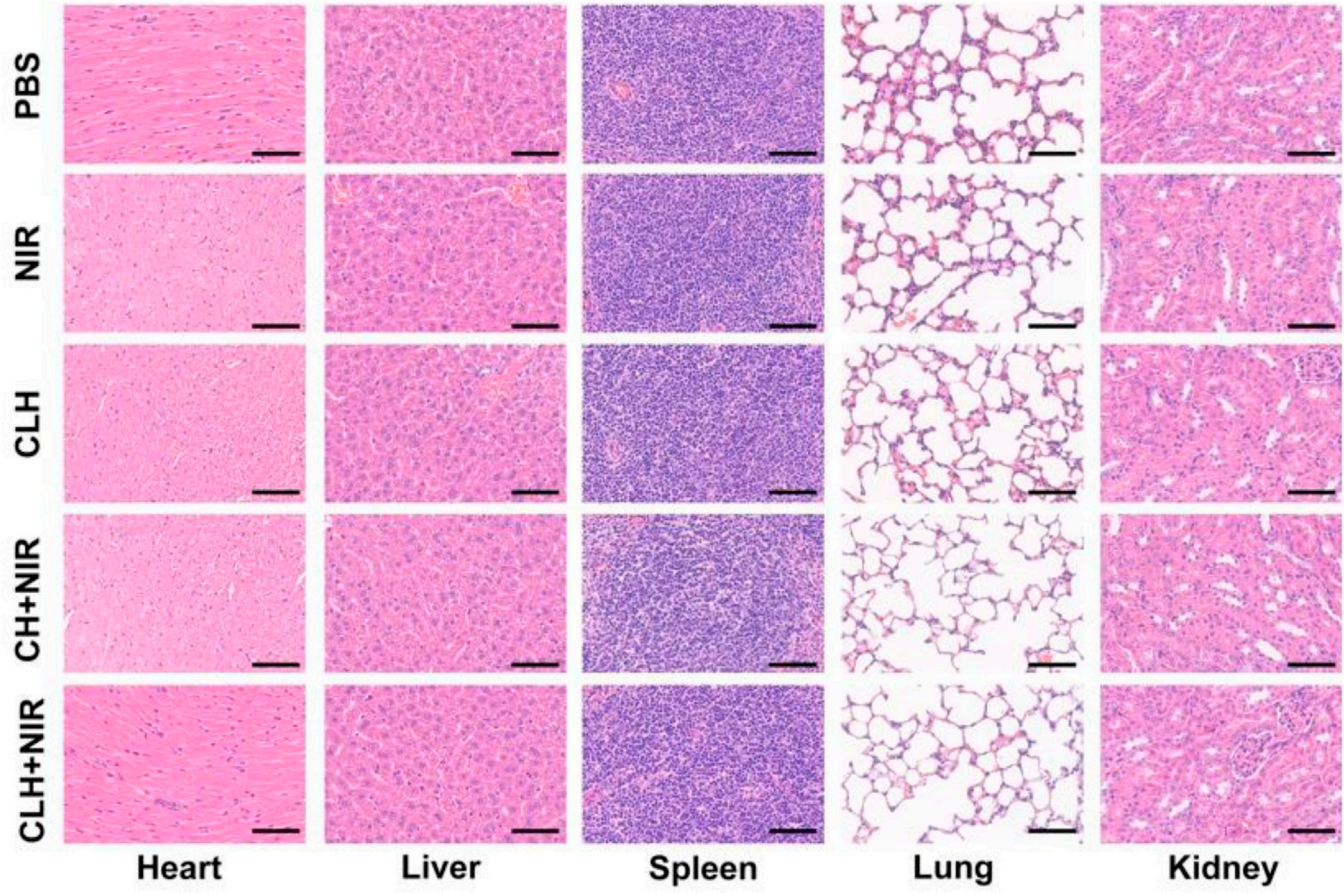
H&E staining of major organs post-treatments. Scale bars: 100 μm.

Prior to probing the *in vivo* treatment effect of CLH, this study initially probed for *in vivo* photothermal effect. Following treatment of A549 murines with PBS and CLH respectively, tumor tissue was irradiated with an 808 nm laser for 5 min, and the increase in tumor temperature was recorded by infrared thermography within different treatment groups. Tumor temperature within CLH group reached 46.6°C within 5 min of 808 nm laser irradiation. Conversely, tumor temperature rose by only 2.3°C following PBS administration within identical irradiation settings (Figure 4A). The skin thickness of nude mice is about 550 μm thick (∼30 μm skin, ∼220 μm leather, ∼300 μm subcutaneously) (Calabro et al., 2011; Byers et al., 2017), so the surface vasculature of the tumor implanted subcutaneously is visible. The 808 nm laser used in this work belongs to the near-infrared region, which can reach the position 5 mm below the skin, and the distance increases with the increase of the spot area (Zhao et al., 2018; Chu et al., 2022). Therefore, PTT can be used for tumor treatment with high clinical application potential. From one perspective, high temperature allow CLH to release CuS NPs and Lap for controlled drug release, while conversely, high temperatures also destroy proteins and selected active substances within tumor tissue, allowing tumor ablation. In addition, CLH is expected to effectively inhibit tumor growth by enhancing the synergistic effect of oxidative stress and glutathione depletion in tumor tissues. Hydrogels containing CuS NPs alone (CH) were prepared as a control group. A549 tumor-bearing nude murines were randomly divided into six groups: 1) PBS; 2) NIR; 3) CH; 4) CLH; 5) CH + NIR; and 6) CLH + NIR. Each treatment group was monitored for tumor volume every 2 days throughout the observation period. As shown in Figures 4B, C, there was no significant inhibition of tumor growth by single application of PBS or 808 nm laser irradiation. In contrast, the CH group had a slight inhibitory effect on tumor growth, which seemed to contradict the results of *in vitro* cytotoxicity tests. In contrast, the CH and CLH groups had a slight inhibitory effect on tumor growth, which seemed to contradict the results of *in vitro* cytotoxicity tests. In fact, hydrogels are decomposed slowly *in vivo* through bioenzyme activity (Qiu et al., 2018; Zhu et al., 2022b; Zhang et al., 2022), and a small amount of CuS NPs released have some inhibitory effect on tumor growth through ROS produced by CDT, while CLH prepared by adding Lap into the hydrogel platform seems to be able to further inhibit tumor growth. The killing effect of CH + NIR group on tumor tissue was further enhanced, which may be because the photothermal effect of CuS under light can accelerate the release of Cu2+, further improve the efficiency of CDT, and thus produce more ROS. However, due to the limited H_2_O_2_ content of the tumor tissue, this resulted in low CDT efficiency and did not completely inhibit tumor growth. Notably, the addition of Lap to the hydrogel platform and the preparation of CLH produced a significant inhibition of tumor proliferation under 808 nm irradiation, since Lap could catalyse the production of H_2_O_2_ through a futile redox cycle, in-turn catalysed by tumor tissue overexpression of NQO1. H_2_O_2_ was further converted to highly toxic hydroxyl radicals through the Fenton-like reaction, leading to a burst of reactive oxygen species in TME, which also validates the ability of CLH + NIR to amplify oxidative stress and complete inhibition of tumor growth. Efficacy following various treatments was further assessed by TdT-mediated dUTP nick-end labeling (TUNEL) staining and Ki-67 staining (Figures 4E–G). In contrast to the control and laser groups, which showed no significant changes in cell status, the TUNEL results showed significant apoptosis/necrosis in both the CLH and CH + NIR groups (Figure 4F). However, the degree of apoptosis and necrosis within CLH + NIR group was significantly higher than for other treatment groups, consistent with the trend of tumor growth. Ki-67 staining was commonly used to detect the proliferation status of cancer cells (Zhu et al., 2021a; Duo et al., 2021), and the results were consistent with the TUNEL results, confirming that the combined photothermal/chemical kinetic synergistic effect of CLH + NIR induced H_2_O_2_ self-supply, in order to achieve reactive oxygen species burst through cascade catalysis and thereby leading to apoptosis of tumor cells. We verified the production of ROS in tumors (Figures 4E and H). The results showed that CLH + NIR group could produce a large amount of reactive oxygen species in the tumor. Therefore, the anti-tumor effect of CLH system was the synergistic effect of ROS and heat. All groups caused no significant body weight loss (Figure 4D). The histopathology changes of major organs including liver, heart, kidney, spleen, lung and brain were collected and investigated post-H&E staining (Figure 5). There were no obvious physiological and morphological changes and inflammatory responses in five groups. And we conducted further blood biochemical analysis (Supplementary Figure S3), all the indicators were normal. This result shows that the health of the mice was not affected after the treatment. The results demonstrated that no organ damage was observed, further confirming the good biocompatibility of CLH.

## Conclusion

In conclusion, our development of injectable thermosensitive hydrogel-loaded hollow copper sulfide nanoparticles and Lap can be used for reactive oxygen species burst in TME and effective tumor therapy. This platform was effective in generating heat under NIL-II radiation, resulting in dissolution of the hydrogel and release of CuS and lap to the tumor site. The acidic TME can accelerate the degradation of CuS NPs. Cu^2+^ can consume the overexpressed GSH in tumor tissues through redox reactions to generate Cu^+^, which further catalyzes H_2_O_2_ to generate cytotoxic hydroxyl radicals. Through this synergistic effect, intracellular ROS levels were significantly increased and oxidative stress was amplified. Simultaneously, under the action of NQO1, the released Lap undergoes a redundant redox cycle to efficiently produce H_2_O_2_, which causes not only direct DNA damage, though also further improve the Fenton-like reaction rate through self-supply of H_2_O_2_, thereby enhancing the apoptotic ability of CDT to tumor cells. This platform, combined with photothermal/chemokinetic synergism, induces H_2_O_2_ self-supply and achieves reactive oxygen species burst through cascade catalysis, thus significantly inhibiting the growth of subcutaneous A549 lung tumors in murines. In the future, this CLH-mediated reactive oxygen burst strategy is expected to enhance traditional tumor therapies. Reactive oxygen species outbreaks within tumor cells often induce non apoptotic death modes such as immunogenic death, pyroptosis, and ferroptosis. Therefore, CLH has great potential in enhancing tumor immunotherapy and reversing tumor chemoradiotherapy resistance.

## Data Availability

The original contributions presented in the study are included in the article/Supplementary Material, further inquiries can be directed to the corresponding authors.
