# Supplementary material for: Injectable thermo-sensitive hydrogel loaded hollow copper sulfide nanoparticles for ROS burst in TME and effective tumor treatment
Source: Front Bioeng Biotechnol. 2023 May 2;11:1191014. doi: 10.3389/fbioe.2023.1191014 (PMC10185793; doi:10.3389/fbioe.2023.1191014)
Supplement: Supplementary file 1 [file DataSheet1.docx]

Experimental Procedures

**Materials and reagents.**

Copper (II) chloride dihydrate (CuCl2·2H2O) and hydrazine hydrate aqueous solution (N2H4·H2O) were purchased from Sinopharm Chemical Reagent Co., Ltd. (China). Sodium sulfide nonahydrate (Na2S·9H2O), poly(vinylpyrrolidone) (PVP K40, Mw = 40,000), 3’-(4-hydroxyphenyl) fluorescein (HPF) and Hydrogen peroxide (H_2_O_2_) fluorescence detection kit were purchased from Sigma-Aldrich. Agarose was purchased from Yare Shanghai. The reduced GSH assay kit was purchased from Nanjing Jiancheng Bioengineering Institute. β-Lapachone (Lap) was purchased from Aladdin. The other reagents used in this work were purchased from Sinopharm Chemical Reagent (China) and Aladdin-Reagent (China).

**Cell culture**

A549 cancer cell line was obtained from the Cell Bank of the Chinese Academy of Sciences and incubated in RPMI-1640 medium supplemented with 10% FBS in a humidified atmosphere at 37℃.

**Preparation and characterization of Copper Sulfide Nanoparticles (CuS)(**[**1**](#_ENREF_1)**)**

Briefly, a CuCl_2_ solution (100 μL) and poly(vinylpyrrolidone) (0.24 g) were mixed in deionized water (25 mL) and magnetically stirred at room temperature (rt). Next, NaOH (pH 9, 25 mL) solution was transferred to the mixture, followed by the addition of hydrazine solution (6.4 μL), which resulted in the formation of a suspension of Cu_2_O spheres. Subsequently, a Na_2_S (320 mg mL−1, 200 μL) aqueous solution was added to the Cu_2_O suspension, stirred at 60 °C for 2 h before cooling to rt, and centrifuged (11 000 rpm, 10 min), followed by washing with deionized (DI) water (twice) to obtain CuS. The morphology structures of CuS were detected by the TEM (JEOL-2100). UV-vis spectra of samples were recorded by the UV-vis spectrophotometry Lambda 35 (Perkin-Elmer).

**Cu^2+^ release study**

10mL of CLH containing 15mg CuS was added into culture dish. To investigate the stimuli effect of laser irradiation and pH on the release behavior, the release experiment of Cu^2+^ was initially performed with or without 0.5 W/cm^2^ 808 nm laser irradiation under different pH value for 10 min. At appropriate time point, 100 μL of different samples were collected for ICP-AES measurement.

**Preparation and characterization of CLH**

The general protocol for the hydrogel preparation is as follows. The prepared CuS (10mg) and LAP (20μg) were added into 10mL 2% agarose solution to form CLH. Scanning electron microscopy (SEM) images were captured on a Hitachi FE-SEM S4800 instrument with an acceleration voltage of 3 kV.

Rheology experiments were performed on an Anton Paar rheometer. Hydrogel samples of different temperatures were prepared and gently placed on the middle of a 15 mm diameter parallel plate with a proper gap. Dynamic oscillatory frequency sweep measurements were conducted at a 1% strain amplitude. To prevent the evaporation of water, a lid was prepared on the top.

**Photothermal Conversion Ability**

An 808 nm NIR laser (Changchun New Industries Tech.Co., Ltd., Changchun, China) with irradiation powers was used to stimulate the concentrations of CuS (0, 50, 100, 200 ug/mL) in an aqueous medium. The photothermal images of the CuS-based suspensions during laser irradiation were recorded every 30 s using an infrared thermal imaging system. Heating curve of CLH for four cycles at a power intensity of 0.5 W cm^-2^ under 808nm laser was measured by the infrared thermal imaging system.

**LAP release study**

The in vitro LAP release profile from CLH was carried out. 10mL of CLH containing 20 μg LAP was added into culture dish. To investigate the stimuli effect of laser irradiation on the release behavior, the release experiment of LAP was initially performed with or without 0.5 W/cm^2^ 808 nm laser irradiation for 3 min. At appropriate time point, 100 μL of different samples were collected, and an UV-vis spectrophotometer was used to monitor the released LAP content.

***In vitro* anti-cancer effect of CLH**

Typically, A549 cells were incubated in six-well plates at 37 °C with 5% CO_2_ for 24 h; afterward, the culture medium was replaced by new culture medium, cells were incubated with 6 different groups at different CuS concentration: (1) PBS; (2) NIR; (3) CH; (4) CLH; (5) CH + NIR; (6) CLH + NIR. Then, cells in group 2, 5 and 6 were exposed to 808 nm laser radiation (0.5 W/cm^2^) for 5 min. Finally, the viability of A549 cells was determined by a CCK-8 cell cytotoxicity assay. The cell viability was normalized by control group without any treatment.

***In vitro* ROS generation**

ROS generation was also assessed in vitro on A549 cells. The intracellular generation

of ROS was detected utilizing HPF and H_2_O_2_ detection kit. Afterwards, cells were incubated for 6 different groups: (1) PBS; (2) NIR; (3) CH; (4) CLH; (5) CH + NIR; (6) CLH + NIR. The LAP concentration was 2μg/mL in group 4 and 6. Then, HPF or H_2_O_2_ detection kit were added. Then, cells in group 2, 5 and 6 were exposed to 808 nm laser radiation (0.5 W/cm^2^) for 5 min and detected under a fluorescent microscope (IX81, Olympus, Japan). Fluorescence intensity was measured by ImageJ software.

**Detection of Intracellular GSH.**

The commercially available GSH assay kit was used to detect the depletion of GSH. A549 cells were incubated with 6 different group at different CuS concentration: (1) PBS; (2) NIR; (3) CH; (4) CLH; (5) CH + NIR; (6) CLH + NIR. Then, cells in group 2, 5 and 6 were exposed to 808 nm laser radiation (0.5 W/cm^2^) for 5 min. After 12 hours of incubation, the GSH content was measured by employing a commercial colorimetric GSH assay kit. The assay was carried out according to the manufacturer’s instructions. The absorbance of 340 nm was measured by a microplate reader.

**Animal tumor models**

Female BALB/c nude mice aged 4-5 week were purchased from Vital River Company (Beijing, China). 100 μL of A549 cell suspension (1×10^6^ cells per mL) were subcutaneous injected into each mouse to establish the tumor models. The animal experiments were carried out according to the protocol approved by the Ministry of Health in People’s Republic of PR China and were approved by the Administrative Committee on Animal Research of the Shenzhen people’s hospital.

***In vivo* infrared thermography**

To monitor the in vivo photothermal effect, CLH was intratumorally injected into the tumor-bearing mice, and then the tumors suffered from 0.5 W/cm^2^ irradiation for 10 min at 1 h post-injection. PBS injection used as control group.

**Evaluation of intratumoral oxidative stress**

The A549 tumor model was used. The mice were firstly divided randomly into 5 groups (each group included 3 mice): (1) PBS; (2) NIR; (3) CH; (4) CLH; (5) CH + NIR; (6) CLH + NIR. Then, mice in group 2, 5 and 6 were exposed to 808 nm laser radiation (0.5 W/cm^2^) for 5 min. The CuS dose was 20 mg/kg in group 3, 4, 5and 6.The Lap dose was 25 mg/kg in group 4 and 6. The injection method is intratumoral injection. NIR was conducted 1h after the injection. The cryosections were stained with DHE and observed by a confocal laser scanning microscope (CLSM; IX81, Olympus, Japan).

***In vivo* antitumor study**

The mice were firstly divided randomly into 5 groups (each group included 5 mice): (1) PBS; (2) NIR; (3) CH; (4) CLH; (5) CH + NIR; (6) CLH + NIR. Then, mice in group 2, 5 and 6 were exposed to 808 nm laser radiation (0.5 W/cm^2^) for 5 min. The CuS dose was 20 mg/kg in group 3, 4, 5and 6. The Lap dose was 25 mg/kg in group 4 and 6. The injection method is intratumoral injection. NIR was conducted 1h after the injection. Mice body weight and tumor volume in all groups were monitored every 2 days. A caliper was employed to measure the tumor length and tumor width and the tumor volume was calculated according to following formula. Tumor volume = tumor length × tumor width^2^ / 2. After 14 days treatment, mice were sacrificed. Five main organs (heart, liver, spleen, lung and kidney) of all mice were harvested, washed with PBS, and fixed with paraformaldehyde for histology analysis. And the tumor tissues were weighed, and fixed in 4% neutral buffered formalin, processed routinely into paraffin, and sectioned at 4 μm. Then the sections were stained with Ki-7 and TUNEL and finally examined by using optical microscope (BX51, Olympus, Japan) and fluorescence microscope (IX81, Olympus, Japan). Fluorescence intensity was measured by ImageJ software.

**Statistical analysis**

Data analyses were conducted using the GraphPad Prism 5.0 software. Significance between every two groups was calculated by the student’s t-test. *P < 0.05, **P < 0.01, ***P < 0.001.


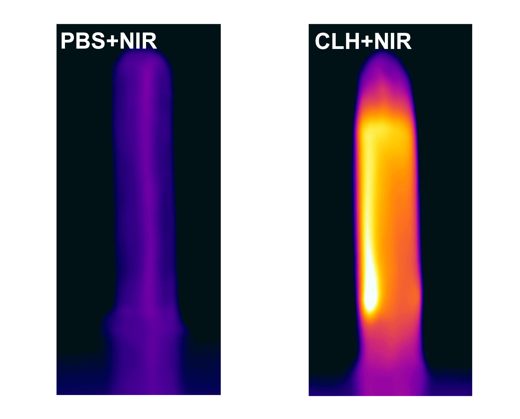


**Figure S1**. Infrared thermal images of the prepared CLH before and after 808 nm laser at 0.5 W/cm2 irradiation for 3 minutes.


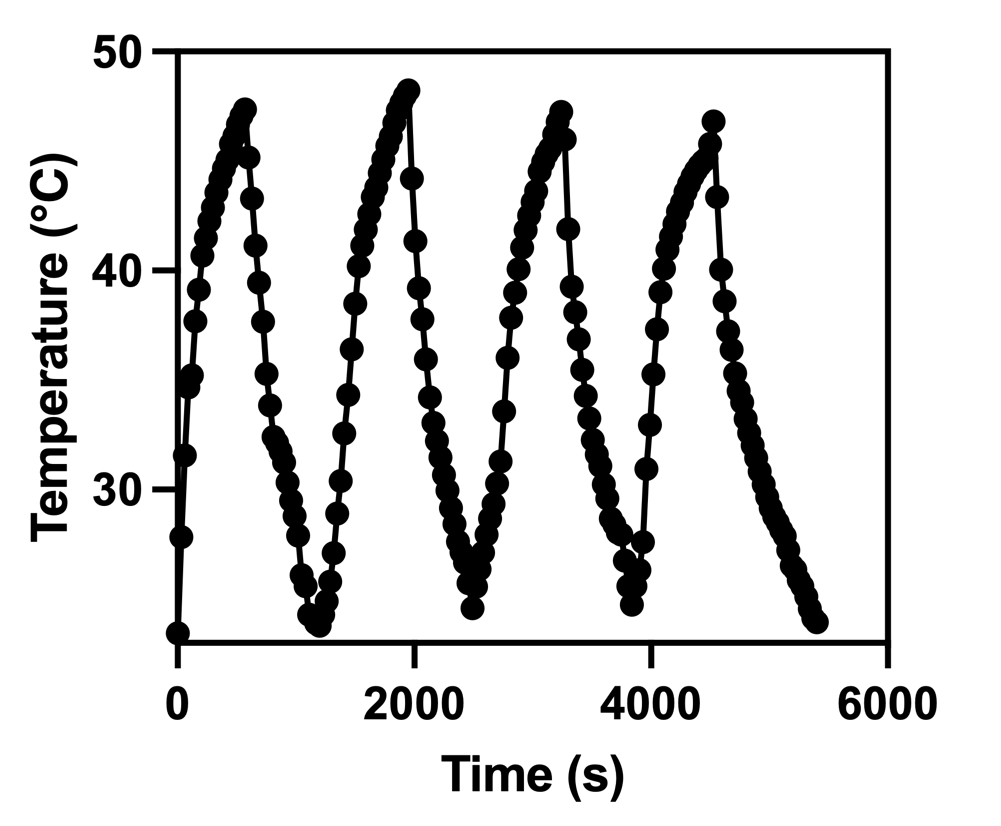


**Figure S2.** Heating curve of CLH for four cycles under 808 nm laser at 0.5 W/cm^2^ irradiation


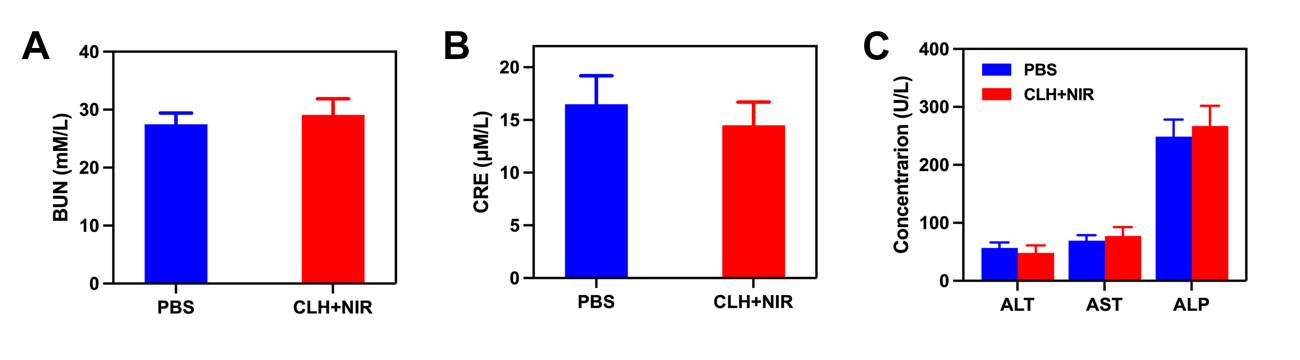


**Figure S3**. Blood biochemical analysis of mice after different treatments.

**References**

1. W. Liu, H. Xiang, M. Tan, Q. Chen, Q. Jiang, L. Yang, Y. Cao, Z. Wang, H. Ran and Y. Chen: Nanomedicine Enables Drug-Potency Activation with Tumor Sensitivity and Hyperthermia Synergy in the Second Near-Infrared Biowindow. *ACS Nano*, 15(4), 6457-6470 (2021) doi:10.1021/acsnano.0c08848
